# Stearic Acid as an Atomic Layer Deposition Inhibitor: Spectroscopic Insights from AFM-IR

**DOI:** 10.3390/nano13192713

**Published:** 2023-10-06

**Authors:** Saumya Satyarthy, Md Hasan Ul Iqbal, Fairoz Abida, Ridwan Nahar, Adam J. Hauser, Mark Ming-Cheng Cheng, Ayanjeet Ghosh

**Affiliations:** 1 Department of Chemistry and Biochemistry, The University of Alabama, Tuscaloosa, AL 35487, USA; ssatyarthy@crimson.ua.edu (S.S.); mhasanuliqbal@crimson.ua.edu (M.H.U.I.); 2 Department of Electrical and Computer Engineering, The University of Alabama, Tuscaloosa, AL 35487, USA; fabida@crimson.ua.edu (F.A.); mmcheng@eng.ua.edu (M.M.-C.C.); 3 Department of Physics and Astronomy, The University of Alabama, Tuscaloosa, AL 35487, USA; rnahar1@crimson.ua.edu (R.N.); ahauser@ua.edu (A.J.H.)

**Keywords:** atomic layer deposition, carboxylic acid, self-assembled monolayers, inhibitor, atomic-force microscopy, infrared spectroscopy, AFM-IR

## Abstract

Modern-day chip manufacturing requires precision in placing chip materials on complex and patterned structures. Area-selective atomic layer deposition (AS-ALD) is a self-aligned manufacturing technique with high precision and control, which offers cost effectiveness compared to the traditional patterning techniques. Self-assembled monolayers (SAMs) have been explored as an avenue for realizing AS-ALD, wherein surface-active sites are modified in a specific pattern via SAMs that are inert to metal deposition, enabling ALD nucleation on the substrate selectively. However, key limitations have limited the potential of AS-ALD as a patterning method. The choice of molecules for ALD blocking SAMs is sparse; furthermore, deficiency in the proper understanding of the SAM chemistry and its changes upon metal layer deposition further adds to the challenges. In this work, we have addressed the above challenges by using nanoscale infrared spectroscopy to investigate the potential of stearic acid (SA) as an ALD inhibiting SAM. We show that SA monolayers on Co and Cu substrates can inhibit ZnO ALD growth on par with other commonly used SAMs, which demonstrates its viability towards AS-ALD. We complement these measurements with AFM-IR, which is a surface-sensitive spatially resolved technique, to obtain spectral insights into the ALD-treated SAMs. The significant insight obtained from AFM-IR is that SA SAMs do not desorb or degrade with ALD, but rather undergo a change in substrate coordination modes, which can affect ALD growth on substrates.

## 1. Introduction

Advances in chip nanomanufacturing have led to the development of integrated circuits (ICs) with sizes below 5 nanometers (nm) [1]. Advanced patterning techniques often employed in state-of-the-art chip fabrication, such as lithography and etching, are becoming more challenging, expensive and time-consuming to cater to the precision required for this top-down approach [1,2]. Area-selective atomic layer deposition (AS-ALD) has been gaining attention as an advanced, bottoms-up approach that can accelerate nanoscale fabrication by potentially reducing the number of lithography steps. AS-ALD is a scalable atomic-scale additive nanomanufacturing method offering the control of not only the film thickness, but also of the spatial locations where the films are deposited. 

ALD is currently used in the semiconductor industry, and a wide range of ALD chemistries, including metals, oxides, sulfides, nitrides, organics, and hybrids, have been developed to that end [2,3,4,5,6,7,8,9]. AS-ALD builds upon ALD, which relies on substrate-site-limited surface reactions, which enable ALD to coat complex three-dimensional structures with atomic-level precision, and of film thickness and composition, on large scales [10,11,12,13,14,15,16,17,18]. AS-ALD gives an additional capability to ALD: it directs ALD to where it is needed through selective surface reactions. ALD has tremendous potential to complement lithography to further reduce the feature size, processing steps, variability, and cost of manufacturing integrated circuits. One approach towards realizing AS-ALD is by selectively passivating surface sites with chemisorbed molecules (i.e., inhibitors), so that ALD growth occurs only on the untreated areas [10,12,14,17,18,19]. The appeal of this method lies in the fact that it requires no special ALD precursors to be designed. The chemisorption of self-assembled monolayers (SAMs) with an inert tail group is used to selectively block out these surface sites. There are two main challenges in the application of SAMs for AS-ALD. Firstly, the choice of inhibitors is small, which have mostly been different variants of organo-silanes, organo-phosphonic acids and organo-thiols [14,17,18,20,21,22,23]. Hence, there is a need for the evaluation of inhibitors based on other functional groups for applications in AS-ALD. Recently, Bent et al. tested the potential of methyl-sulfonic-acid SAMs as ALD-blocking agents [24]. However, other inhibitors of ALD need to be evaluated to further the practical applications of AS-ALD. Secondly, the precise role of the SAM in ALD blocking is also not well understood [16,25]. SAMs lose their ability to block ALD growth with increasing ALD cycles, and it has been hypothesized that either the morphological defects resulting from packing inefficiencies lead to localized nucleation, or that trapped ALD reactants lead to SAM degradation, eventually resulting in the loss of ALD blocking [12,15,25]. However, direct spectroscopic measurements of SAMs upon ALD have been lacking. It is not well known to what degree SAMs undergo degradation, if any, upon ALD nucleation, and how that correlates with ALD growth. This arises in part from the limited capability in analyzing the chemistry of SAMs at the nanoscale before and after ALD cycles. Infrared (IR) spectroscopic techniques have been widely used in conjunction with ALD [12,26,27,28,29,30,31,32]; however, conventional Fourier-Transform Infrared (FTIR)-based approaches are limited in sensitivity and not always suitable for probing monolayers. Furthermore, metallic substrates that are not transparent to IR radiation are often used in ALD, thus limiting the applications of transmission-based FTIR methods. Infrared reflection–absorption spectroscopy (IRRAS or RAIRS) is more suited for studying thin films on reflective substrates like metals [33,34,35,36]. However, surface selection rules apply to IRRAS measurements, which selectively amplify certain spectral components depending on their dipole moment orientation [36,37,38]. These selection rules can also vary between substrates, posing additional challenges to using IRRAS for characterizing ALD-treated SAMs. 

**Figure 1:**
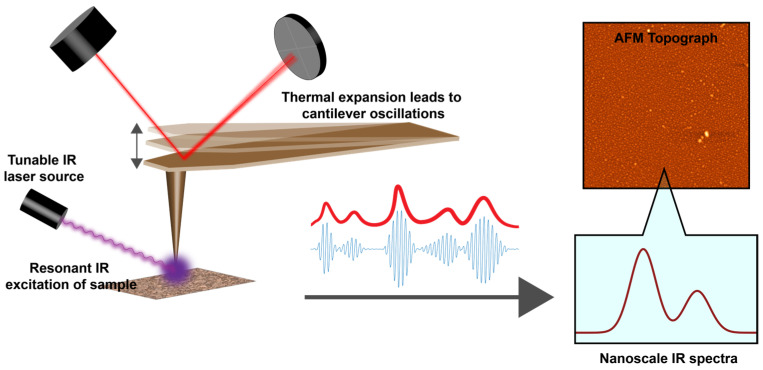
Schematic of AFM-IR operation. Infrared absorption results in photothermal expansion of the sample, leading to cantilever oscillations, which is sensed by the AFM photodetector. Measuring the oscillation amplitude as a function of the excitation wavelength yields a spectrum localized to nanoscale spatial dimensions.

In this paper, we investigate the viability of carboxylic acids as potential inhibitors for zinc oxide (ZnO) ALD on copper (Cu) and cobalt (Co) substrates, with an aim to addressing the above challenges. Specially, stearic acid (SA) SAMs are studied, which have a carboxylic acid headgroup that binds to the metallic substrate, with an 18-carbon chain terminated with a methyl (-CH3) group. In semiconductor manufacturing, the back-end-of-line (BEOL) fabrication step involves the formation of interconnects between components such as transistors, capacitors, etc. Cu has been widely used as interconnects in the semiconductor industry. However, Cu interconnects can suffer from reliability issues arising from the diffusion of Cu atoms [39,40]. As a result, other metals, such as Co and Ru, have been explored as alternatives to Cu interconnects [41,42,43,44]. Our choice of substrates, hence, covers two of the more commonly used metals for interconnects in chip manufacturing. Studying the monolayers on both Cu and Co substrates also allows for the assessment of their relative ALD-blocking abilities. An ideal SAM should be able to inhibit ALD nucleation on both substrates to some degree to be suitable for applications in chip fabrication. Furthermore, ALD inhibition by phosphonic acids on Cu and Co substrates has been demonstrated previously [10,17], and we have chosen to study SA SAMs on the same substrates to allow for the direct comparison of SA with other ALD inhibitors. To obtain spectroscopic insights into the SAM structure before and after ALD, we use AFM-IR, which is an emerging technique that combines atomic-force microscopy (AFM) with infrared (IR) spectroscopy [45,46,47,48,49], and offers monolayer sensitivity [49,50], ideal for characterizing how the chemistry of SAMs is affected by the ALD process. In AFM-IR, the photothermal expansion induced by IR absorption is sensed by an AFM cantilever, which leads to high sensitivity combined with nanoscale spatial resolution (Figure 1). This allows for the better identification and interpretation of spectra. In conventional spatially averaged spectroscopy, there is no way to discern if a particular spectral feature arises from localized defects/impurities or is evenly distributed through the specimen. AFM-IR circumvents this limitation and offers insights into the spatial distribution of chemistry. Furthermore, since AFM-IR does not directly measure infrared radiation, but rather the effect of infrared absorption through an AFM cantilever, it is free from scattering artifacts that can pose additional challenges. The capability of AFM-IR to probe buried interfaces has recently been demonstrated [51], making it particularly relevant for SAMs underlying metal-oxide layers, as is expected upon ALD nucleation.

We show that SA can block ALD growth on Cu and Co for up to 25 and 50 cycles, respectively. Using AFM-IR, we demonstrate that the monolayer does not degrade upon the deposition of ZnO; suggesting that the breakdown of the SAM is not the main cause behind ALD nucleation and the loss of selectivity. Furthermore, we find that the SA-metal coordination changes with increasing ALD cycles, which points to chemical insights underlying ALD inhibitions by the SAMs. Our results demonstrate that carboxylic acid SAMs can be optimized as suitable ALD inhibitors due to their stability during ALD processing conditions.

## 2. Materials and Methods

### 2.1. Materials

Stearic acid (SA) (≥97%, Sigma-Aldrich, St. Louis, MO, USA) was used as a surface passivation molecule. Ethanol (200 proof, ≥99.5%, Sigma-Aldrich) was used as the solvent for dissolving SA. Prime-grade silicon (Si) wafers (Platypus Technologies, Fitchburg, WI, USA) were used as the supporting substrate for metal-layer coating. ALD precursors were diethylzinc (DEZ) (98%, Strem Chemicals, Newburyport, MA, USA) and deionized water (DI H_2_O). The carrier and purging gas for the ALD process was ultra-high-purity nitrogen (99.999%, Airgas, Radnor, PA, USA).

### 2.2. Sample Preparation

The Cu and Co films, with a thickness of 400 nm, were deposited using sputtering. The deposition process was carried out using an ultra-high vacuum, off-axis, DC magnetron sputter beam epitaxy system (AJA International Inc., Scituate, MA, USA) on a silicon substrate in an Ar atmosphere with a pressure of 5 mTorr. The base pressure of the system was 5 × 10^−9^ Torr, and the substrate temperature was maintained at 100 °C during the deposition process. Prior to depositing the Cu/Co film, a 10 nm aluminum adhesion layer was grown on the Si substrate. The growth process involved maintaining uniform substrate rotation at 30 rpm, and the growth rate was controlled using a quartz crystal microbalance (QCM). The Co and Cu substrates were washed 3 times with ethanol, dried under a gentle stream of nitrogen and cleaned by UV ozone (ozone cleaner, Ossila, Sheffield, UK) for 1 min. All the substrates were then kept under vacuum to prevent oxidation in air. An amount of 1 mM SA in ethanol solution was made for fabricating SA monolayers on these substrates. Substrates were fully immersed inside the SA solution for 48 h. Substrates were subsequently removed from the SA solution, rinsed in ethanol to remove any unwanted physisorbed material, and dried under nitrogen. Substrates were stored under vacuum conditions prior to measurements. 

### 2.3. Atomic Layer Deposition on Monolayer Substrates

A stainless-steel boat with flat bottom was used as the ALD sample holder for the substrates. The precursor for the ZnO ALD was DEZ, and the quenching medium was DI H_2_O. The ALD reaction temperature was set at 100 °C. The sequence followed for each ALD cycle was 400 s of pre-purging, 1 s of precursor (DEZ) pulse (with a transient pressure increase of ~0.2 Torr), 15 s of N_2_ purging, 1 s of DI H_2_O dose (with a transient pressure increase of ~0.2 Torr), and 45 s of N_2_ purging. The processing pressure for ALD was ~1 Torr, with a flow of 250 sccm of N_2_.

### 2.4. Characterization of Monolayers

X-ray photoelectron spectroscopy (XPS) measurements were performed using a VersaProbe II instrument with monochromatized Al Kα X-ray (1486.6 eV) operated at 24.5 W and a pass energy of 187.85 eV with a detection limit of 0.1% (Advanced Materials Characterization Facility, University of Alabama–Birmingham) to quantify the amount of self-assembled monolayers (SAMs) adsorbed on the surface and the amount of atomic layer deposition (ALD) growth on the surface. XPS data were analyzed using the Casa XPS software package. AFM-IR experiments were conducted using a Bruker NanoIR3 system, which integrates an AFM instrument with a quantum cascade IR laser (MIRCAT, Daylight Solutions). All AFM and IR imaging experiments were performed in contact mode at ambient temperature, with the humidity kept at ~5% or lower by dry-air purging. AFM-IR images were recorded at a resolution of 500 pixels × 500 pixels. The AFM images were processed using Gwyddion software (version 2.62). The IR spectra were acquired at a resolution of 2 cm^−1^, and denoised with a (2,5) Savitky–Golay filter and a 3-point moving average filter. All spectra were normalized to the maximum intensity to mitigate any variations in the signal due to changes in the tip–sample interactions. The spectra were processed using the MATLAB software package (version R2023a, MathWorks, Natick, MA, USA). All data were plotted using MATLAB.

## 3. Results and Discussion

**Figure 2:**
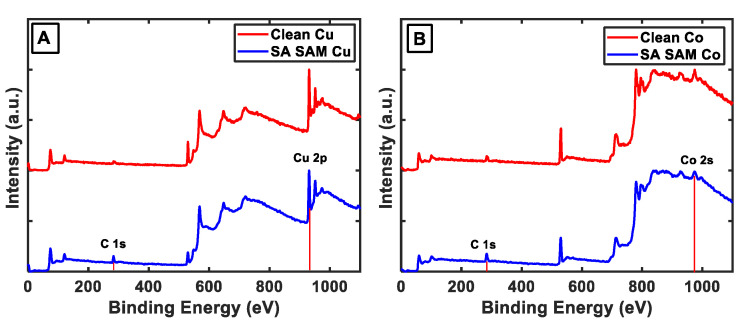
Comparison of XPS IR spectra of bare and SA-treated substrates. (**A**) Cu, and (**B**) Co. The C 1s, Cu 2p and Co 2s peaks are indicated. The increase in the C 1s peak indicates SAM formation on the substrates.

**Figure 3:**
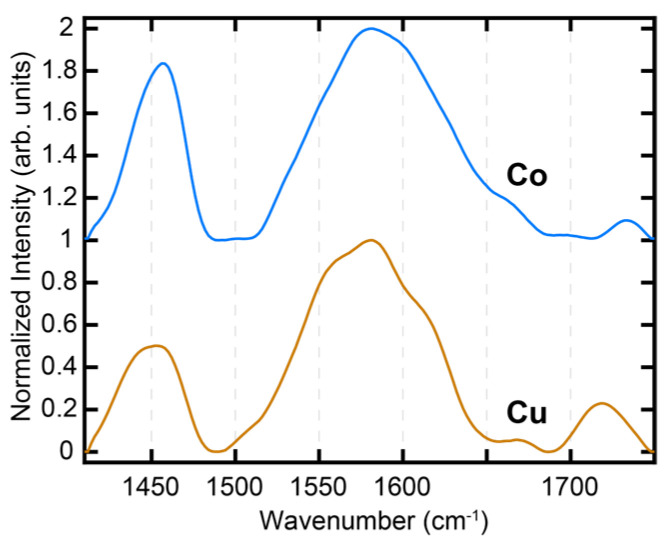
AFM-IR spectra of SA-treated Cu and Co substrates. The spectra are the average of data acquired from multiple spatial locations. The spectra have been normalized for clarity.

To verify the adsorption of SA on the metal/metal-oxide substrates, we first performed X-ray photoelectron spectroscopy (XPS) measurements. Figure 2A,B shows the XPS spectra of the Cu and Co substrates with and without the SA treatment. In both cases, we can observe an increase in the intensity of the C 1s peak at 284.5 eV, which is associated with C-C, from the untreated, bare substrate to that treated with SA. This is consistent with the formation of monolayers on the substrates. To characterize the chemistries of the SAM-treated substrates, AFM-IR experiments were performed, which also served as validation for the sensitivity of AFM-IR towards the SAM vibrational signatures. AFM-IR measurements were performed on a 5 µm × 5 µm area of each of the SA SAM-treated sample (Cu and Co). The morphology of the area was first characterized with an AFM topograph, and subsequently IR spectra were acquired from different spatial locations from within this area. The average spectrum recorded for each substrate in the spectral range of 1410–1750 cm^−1^ is shown in Figure 3. The spectra have been normalized for clarity. Both the Co and Cu samples show prominent peaks at ~1460 cm^−1^, which arose from the -CH_2_ bending /scissoring modes of the stearic acid carbon backbone and the symmetric stretch of the carboxylate headgroup [52,53]. Additionally, both samples exhibit a broad intense peak at ~1570 cm^−1^, which arose from the asymmetric stretching mode of the carboxylate headgroup [52,53,54], and shoulders/peaks at ~1660 cm^−1^ and 1730 cm^−1^. The carboxylate peak for both Cu and Co is asymmetric, indicating the possible presence of the underlying substructure having arisen from different coordination conformations of SA on the metals. We discuss the structural implications of the spectra in detail later in the manuscript. The spectra, taken together, clearly indicate that AFM-IR had the desired sensitivity towards mapping the chemistry of the metal–SA interface, and can, hence, be employed to evaluate the change in the same upon ALD treatment. It should be noted in this context that Water Contact Angle (WCA) measurements are often used for the assessment of SAM formation on substrates. However, WCA measurements reflect the change in surface hydrophobicity and do not provide any directly quantifiable chemical information; as a result, we relied on XPS and AFM-IR for the chemical characterization of the SA SAMs.

**Figure 4:**
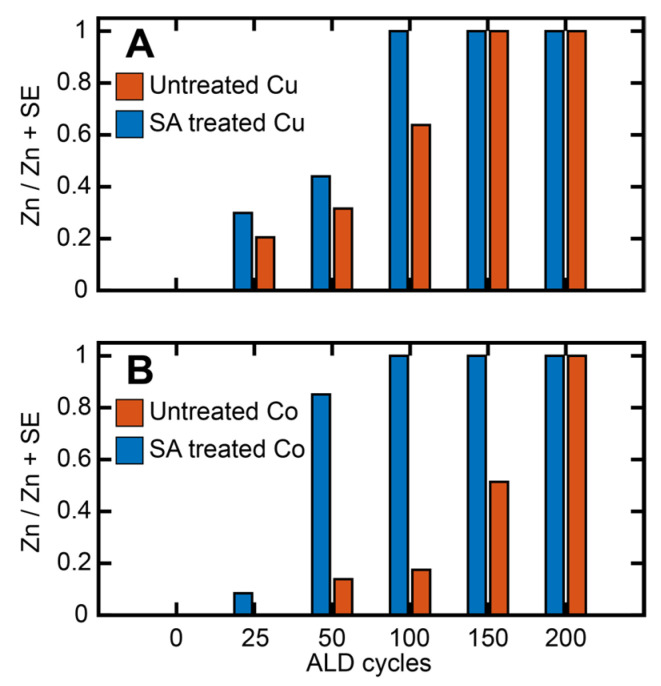
Zn/(Zn + Surface Element) values after different ALD cycles obtained from XPS composition analysis for (**A**) untreated Cu and SA-treated Cu, and (**B**) untreated Co and SA-treated Co.

**Figure 5:**
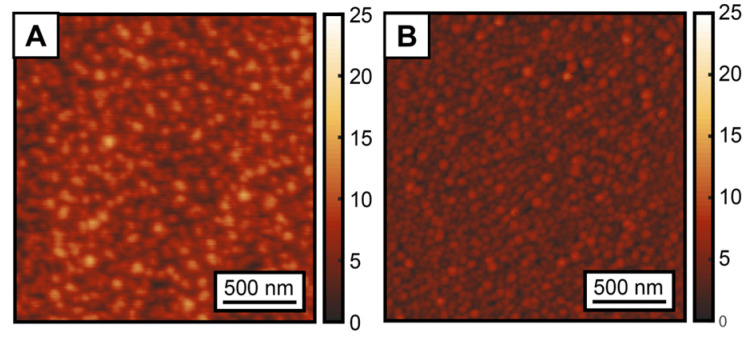
AFM topographs of (**A**) SA-treated Cu and (**B**) SA-treated Co. Each image corresponds to a 2 µm by 2 µm area.

To explore the role of ALD conditions and nucleation on the carboxylate monolayer, ZnO ALD was performed on SA-treated and clean Cu and Co substrate for 25, 50, 100, 150 and 200 cycles. Bent et al. previously demonstrated that ALD nucleation initiates on the Co and Cu substrates with octadecylphosphonic acid (ODPA) SAMs between 25–100 cycles [17]. The tail groups for both ODPA and SA are identical, namely -CH_3_, and a similar nucleation behavior was, thus, expected for the SA SAMs studied here, which dictated our choice of the ALD cycles. X-ray photoelectron spectra (XPS) were acquired on the SAM-treated and untreated metal surfaces after increasing cycles of ZnO ALD to assess ALD nucleation. XPS composition analysis was performed by calculating the value of the following ratio: $\theta =\frac{Zn}{Zn+SE}$, where SE corresponds to the non-SAM surface element (i.e., Cu or Co) [16,17]. θ essentially corresponds to the atomic abundance of Zn relative to the substrate metal SE after a different number of ALD cycles. By definition, the value of θ can range from 0 to 1; θ = 0 represents the absence of Zn on the sample substrate, indicative of no nucleation of ZnO on the surface, while θ = 1 implies that the XPS elemental composition exhibits only the presence of Zn but no surface material, indicating the formation of a ~10 nm of Zn coating on the surface, thick and conformal enough to block the escaping electron from the SE. Figure 4 shows a comparison of the θ value, as determined using XPS compositional analysis, on the bare and SAM-treated Co and Cu substrates after 0, 25, 50, 100 and 200 cycles of ZnO ALD. Without the SA treatment, we observe gradual ZnO growth on Co and Cu with a nucleation time ≤25 cycles. On SA-treated Cu, we observe ALD nucleation also after 25 cycles; however, the theta value is lower in comparison to bare Cu, indicating some inhibitory effect of the SAM. This is in agreement with previous studies on ODPA SAMs [17]. Very recently, López-González et al. demonstrated a similar ALD-blocking ability of SA on Cu [55], which further validates our findings. Stearic acid-treated Co exhibited better ALD blocking, and no ZnO growth was observed until 50 cycles. After 100 cycles, we observed a significant amount of ALD growth on SA-treated Cu, and after 150 cycles, we had a dense layer of ZnO that fully blocked the photoelectrons from the underlying Cu, as reflected by the value of theta reaching unity. SA-treated Co inhibits the significant growth of ZnO compared to untreated Co for up to 150 cycles; however, at 200 cycles, we observe a θ value of 1, indicating the formation of a dense ZnO layer. The results indicate that the adsorbed stearic acid SAMs on Cu and Co can block ZnO ALD nucleation for 25 and 50 cycles, respectively. It should be noted that the inhibition of ALD nucleation is a complex function of multiple parameters, such as monolayer packing, substrate quality and substrate fabrication conditions [10,16,17]. The focus of our work was not to identify the best set of parameters that lead to maximal ALD inhibition by SA SAMs. However, the above results, nonetheless, confirm that SA monolayers can be a viable alternative to ODPA SAMs that are typically used for ALD blocking. The results also suggest that the SA SAMs on Co can block ALD nucleation better than the SA SAM on Cu. This is consistent with previous reports using ODPA [17], where a better ALD blocking ability of Co over Cu was observed. The surface morphology of the substrates is usually identified as one of the main components affecting ALD growth. To verify if this was applicable to the differences observed between Co and Cu, we calculated the surface roughness of the SAMs on each of the substrates from the AFM topographs (Figure 5). The RMS roughness of the SAM surface on Co was determined to be 1.3 nm, while on the Cu, the roughness was 2.1 nm. This variation in surface roughness agreed with the difference in ALD nucleation times for Co and Cu. However, the roughness variations were small, and, hence, it is possible that additional factors could be at play behind the observed differences in the ALD blocking of the SAMs. One possible factor that may have affected these results is the diffusion of copper atoms across the Si/SiO_2_ interface, which can alter the monolayer integrity and, thus, contribute to ALD nucleation. However, the diffusion of copper across dielectrics has been shown to have onset temperatures typically higher than 100 °C, which was the temperature of our ALD reactor [56,57,58,59]. Hence, it is unlikely that the diffusion of copper significantly affected our observations. The optical images of the bare Cu substrates after heating at 100 °C for 1 h, shown in Figure S1, do not exhibit any changes to film quality. Additionally, it should be noted that Cu was not directly deposited onto Si, but onto an adhesion layer of Al, which has been shown to be effective towards limiting Cu diffusion [60]. The thickness of the Cu film was 400 nm, and even if there was some diffusion of Cu atoms into Si, it is unlikely that this distorted the structure of the SAM, which was not at the Cu/Si interface. Also, its coordination to SAM should also limit the diffusion of Cu atoms to some degree. Furthermore, other studies that have employed Cu in ODPA SAM substrates [10,17,20] for ALD have not reported any evidence of Cu diffusion altering ALD nucleation. We therefore discard this possibility as a contributing factor to ALD growth on SAMs on Cu substrates. For ODPA monolayers, differences in ALD blocking abilities have been attributed to the difference in the Lewis acidity between Co and Cu [17], which leads to the different stability of metal–ligand bonds and consequent variations in the packing density of the monolayer. However, this has only been postulated, and to the best of our knowledge, any differences in the binding of ALD-blocking ligands to metal substrates and changes thereof upon ALD growth have not been tested. It is also not well understood if ALD nucleation correlates with changes in monolayer chemistry or its degradation. To identify any possible variations in SA coordination to Co and Cu substrates and their changes during ALD, we therefore employed AFM-IR spectroscopy.

**Figure 6:**
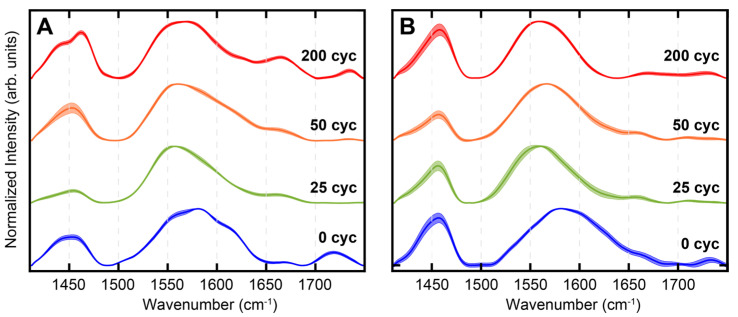
AFM-IR spectra of SA-treated substrates after 0, 25, 50 and 200 cycles of ALD. (**A**) SA on Cu, and (**B**) SA on Co. The shaded area represents the spectral standard deviation. The spectra have been normalized for clarity.

**Figure 7:**
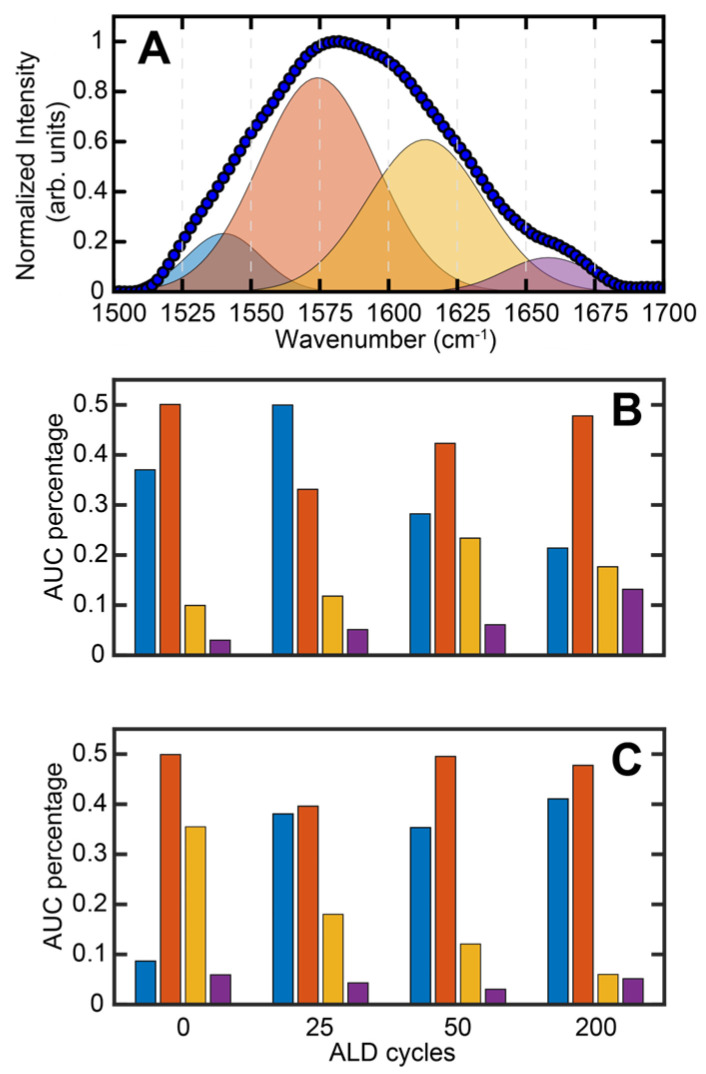
(**A**) Spectral fit of SA on Co (0 cycles). Percentage AUC values, as determined from the spectral fits, for each of the sub-bands are shown for (**B**) SA on Cu and (**C**) SA on Co for 0, 25, 50 and 200 ALD cycles. The bars in (**B**,**C**) are color coded to the spectral bands shown in (**A**).

**Figure 8:**
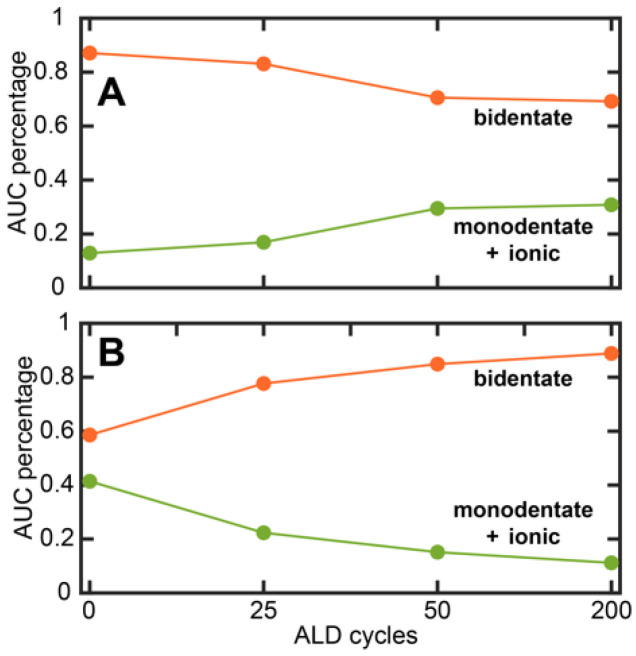
Evolution of the distribution of coordination states for SA SAMs on (**A**) Cu and (**B**) Co. The AUC percentages shown in Figure 7 have been used to calculate the overall percentage of the coordination states.

AFM-IR spectra were acquired on SA-treated Co and Cu after 25, 50 and 200 cycles of ALD to gain insights into SA coordination to the substrate and how it is affected by ALD nucleation. The spectra are shown in Figure 6A,B. Representative AFM images are shown in Figures S2 and S3. For clarity, the spectra corresponding to no ALD (i.e., 0 cycles) are also shown for each substrate. For each sample, approximately 36 spectra were averaged from multiple spatial locations. We observed that the spectral bands from SA persisted throughout the ALD process, which suggests no major degradation/desorption of the SAM at the ALD processing temperature of 100 °C. This implies that monolayer degradation/desorption may not be the major mechanism behind the loss of ALD-blocking ability. This is an important result, since the role of SAM degradation on ALD has rarely been studied in-depth. The deoxygenation of carboxylic acids on metal substrates can also serve as a mechanism of monolayer degradation; however, these processes usually require temperatures higher than that used for the ALD treatment in this work [61,62,63,64]. Furthermore, any decomposition of the SAM will be reflected in the IR spectra; since persistent carboxylate bands from SA are observed before and after ALD, we, therefore, rule out any decomposition of the SAM. The persistent presence of SA spectral markers throughout ALD indicates the robustness of SA SAMs with respect to tolerating the ALD conditions. Another significant observation that can be made from the spectra is their homogeneity: the spectral features at any given ALD cycle on either substrate do not exhibit significant spatial variation, as evidenced by their small standard deviations. This indicates that the monolayer chemistry, as reflected in the spectra, is homogeneous. This also highlights one of the important capabilities of spatially resolved measurements like AFM-IR: in absence of spatial resolution, it is difficult to discern true spectral features and variations thereof from artifacts from localized impurities. The broad spectral features from ~1500–1700 cm^−1^ can be attributed to the carboxylate asymmetric stretching vibration based on prior reports [52,53,54]. The band between 1400 and 1500 cm^−1^ arises from the -CH_2_ bending vibrations of the stearic acid backbone but can also contain contributions from the carboxylate symmetric stretch, and as a result is more difficult to interpret. We also observe an additional band at ~1730 cm^−1^ for some of the spectra. This band can be attributed to the -COOH vibration of SA. Upon the coordination to acidic metal substrates, the major fraction of stearic acid converts to the anionic carboxylate form, which has also been demonstrated in prior studies [65,66]. However, a small fraction of the acid form can remain, as evidenced by the spectra in Figure 6. Interestingly, the -COOH mode in pure stearic acid appears at a lower frequency, at ~1700 cm^−1^. The shift of this mode to higher wavenumbers indicates that it likely does not represent free uncoordinated acid. This frequency shift can arise from the change in the local environment: it is well known that vibrational frequencies of carbonyl modes are sensitive to the local electrostatic field [67,68], which is expected to be significantly different in an ordered monolayer on a metallic substrate compared to an in bulk solvent. Hence, the shift in vibrational frequency is not unexpected. Previous reports have also identified this band and attributed it to the monodentate coordination mode of the carboxylic acid moiety [65]. However, we did not observe any particular trends in the intensity of this band with ALD, and, therefore, chose to focus on the carboxylate vibration between 1500 and 1700 cm^−1^ for spectral analysis. For SA on both Co and Cu, we observed a broad spectrally asymmetric peak shape for the carboxylate mode, indicating presence of underlying sub-bands. We further observed a change in the peak shape with ALD, indicating a change in the relative distribution of these sub-bands. Additionally, for SA on Cu, we observed a peak at ~1660 cm^−1^ that becomes more prominent with ALD. To gain further insights into the chemistry of SAM coordination, we deconvoluted the spectra using band fitting. It is known that carboxylate groups can bond to metals through monodentate and bidentate coordination [52,53,54,65]. Bidentate coordination can be further categorized into bridging and chelating modes. In addition, there can also be electrostatic/ionic interaction between the physisorbed carboxylate moiety and the metal site, with no bond between the two. Therefore, we fit the carboxylate band to four sub bands corresponding to the above modes of interaction/coordination. A representative spectral fit, corresponding to the spectrum of SA on Co without ALD treatment (i.e., 0 cycles), is shown in Figure 7; the other fit results are shown in the Supporting Information (Figure S4). From the fitting analysis, each spectrum can be deconvoluted as a linear combination of four bands at ~1545 cm^−1^, ~1580 cm^−1^, ~1610 cm^−1^ and ~1660 cm^−1^. It is known that bidentate coordination results in lower vibrational frequencies compared to physisorbed/ionic carboxylate, which does not form a coordination bond with the metal centers, whereas monodentate coordination shifts the frequency to higher wavenumbers [53,54]. Therefore, we tentatively assigned the bands to ~1545 cm^−1^ and ~1585 cm^−1^ bands to bidentate coordination, the ~1610 cm^−1^ band to physisorbed/ionic and the ~1660 cm^−1^ band to monodentate carboxylate moieties, respectively. While such assignment was consistent with previous reports on the infrared spectroscopy of carboxylates, the frequencies of each coordination mode can vary depending on the metal and the carboxylate tail group; therefore, an unequivocal spectral assignment was not possible. Nonetheless, the fitted components still represented different binding modes of SA to the substrates with varying degrees of coordination strength. The less stable (monodentate and physisorbed/ionic) coordination states are expected to exhibit a higher vibrational frequency compared to the more stable bidentate states. The spectral components, irrespective of their precise origins, are, thus, reflective of the surface chemistry of the SAMs after different ALD cycles. From the fitted spectra, we calculated the area under the curve (AUC) for each of the sub-bands, which reflect the population of each spectral component. The percentage AUC of the bands for different ALD cycles on Co and Cu is shown in Figure 7B,C. From this analysis, we can conclude that with ALD, there is a change in the monolayer chemistry, specifically in the coordination of the carboxylate unit to the metal centers. For Cu, we observed a gradual increase in the population of the 1660 cm^−1^ component with increasing ALD cycles, which indicates that upon ALD, the bonding between the SAM and the substrate changes and results in a relative increase in a more weakly bound monodentate population. Interestingly, for Co, we did not observe this trend. Instead, we observed an increase in the population of the 1545 cm^−1^ band with increasing ALD cycles. Given that this band arises most likely from the bidentate coordination of SA to Co, this suggests that with ALD, while the bonding SA-Co bonding does change, it does not shift towards a weaker, monodentate SA population, pointing to a fundamental difference in how the SA SAMs on each substrate respond to ALD treatment. This can be better visualized when grouping the spectral components into bidentate and monodentate/ionic groups. As shown in Figure 8, the relative abundance of monodentate and ionic species increased for Cu while the bidentate population decreased. For Co, the exact opposite trend was observed. Essentially, for Cu, ALD leads to a gradual increase in more weakly bound coordination states, whereas for Co, the population of more strongly bound states increases. This can be rationalized by taking into account the oxophilicities of Cu and Co. The oxophilicity of metals is related to their electronegativity and effective nuclear charge: essentially, less-electronegative metals bind more strongly to highly electronegative oxygen [69,70]. The varied affinity of different metals towards oxygen is well known and is considered pivotal to a wide range of processes [70]. Co is more oxophilic than Cu, which may explain why the SAMs on Co do not undergo a significant change towards more weakly bound coordination states. This structural evolution of the SAM with ALD correlated with the oxophilicity of the substrate has never been demonstrated before. We aim to test the generality of this finding through testing ALD nucleation on other transition-metal substrates in future work. For ODPA, it has been shown that the distribution of coordination states is an important factor for the ordering of the alkyl chains, which in turn is key to the structural order of the monolayers [71]. Since SA and ODPA share the same alkyl tail, similar effects of headgroup coordination on the monolayer packing can, thus, be expected. For Cu and Co, we observed the different reorganization of the SAM coordination with ALD, which could, thus, be a factor, in addition to morphology, that facilitates/inhibits ALD growth. Taken together, the above results provide new fundamental insights into the chemical evolution of the SAMs with ALD and how this could be related to their blocking abilities, and highlights the importance of exploring SAM chemistry as a metric for the better optimization and design of ALD inhibitors.

## 4. Conclusions

In summary, we have assessed in this work the potential of carboxylic acid monolayers as ALD inhibitors for applications in AS-ALD. We investigated the ZnO ALD growth of SA-treated substrates and demonstrated that SA inhibits ALD nucleation for up to 25 and 50 cycles on Cu and Co, respectively. These numbers are consistent with other commonly used ALD inhibitors like phosphonic acid, and, thus, confirm stearic acid as a viable option to SAM-mediated ALD blocking. Using AFM-IR spectroscopy, we further explored the change in the SAM chemistry with ALD cycles. We identified that while there is no major degradation of the SAMs on either substrate, the distribution of the coordination states of SA on Cu changes with ALD to having a higher relative proportion of monodentate. This could potentially facilitate ALD growth by altering the monolayer packing. In contrast, the SA on Co does not exhibit the same evolution with ALD, which indicates a potentially different underlying mechanism of ALD nucleation on this substrate. One of the key factors holding back applications of SAM-based AS-ALD has been the limited number of SAMs that can be used for ALD blocking, and a concurrent lack of understanding of how the SAM chemistry changes upon ALD growth. Our work has addressed both the above challenges by demonstrating the viability of stearic acid SAMs for ALD blocking, and the role of the SAM chemistry towards ALD growth. These results pave the way for not only the exploration and optimization of carboxylic acids as a new class of monolayer forming molecules for application towards AS-ALD, but also for the applications of AFM-IR as a suitable technique for probing the chemistry of buried interfaces, particularly in the context of ALD. While our choice of Cu and Co substrates allowed for the comparison of SA SAMs with previous studies on ODPA, the results reported are not necessarily limited to only these substrates. SA is expected to form SAMs on other metallic surfaces as well, and, therefore, exhibit the same ALD-blocking ability observed here. The key insights emerging from this work are that a) SA SAMs can inhibit ZnO ALD nucleation, and b) the eventual ALD growth of ZnO on SA SAMs is not correlated with the degradation of the SAM but rather with the change in coordination of the SAM to the substrate. Furthermore, the latter appears to be dependent on the Lewis acidity and/or oxophilicity of the metallic substrate. These insights into the structural rearrangement of SAMs upon ALD are unique, and we aim to expand on these studies to understand ALD induced changes in other SAMs, as well as optimizing SA SAMs for AS-ALD, in future work.

## Data Availability

All the data required to reach the conclusions made in this report have been provided in the manuscript and Supporting Information. Additional data are available upon reasonable request.

## Supplementary Materials

The following supporting information can be downloaded at: https://www.mdpi.com/article/10.3390/nano13192713/s1, Figure S1: Images of of copper substrates before and after heating, Figures S2 and S3: AFM images of ALD-treated SAMs on Cu and Co after 25, 50 and 200 cycles, respectively, Figure S4: Fits to AFM-IR spectra of SA-treated Cu and Co after different ALD cycles.

## References

[1] Liddle J.A., Gallatin G.M. (2016). Nanomanufacturing: A Perspective. Acs Nano.

[2] Mackus A.J.M., Bol A.A., Kessels W.M.M. (2014). The Use of Atomic Layer Deposition in Advanced Nanopatterning. Nanoscale.

[3] George S.M. (2010). Atomic Layer Deposition: An Overview. Chem. Rev..

[4] Miikkulainen V., Leskela M., Ritala M., Puurunen R.L. (2013). Crystallinity of Inorganic Films Grown by Atomic Layer Deposition: Overview and General Trends. J. Appl. Phys..

[5] (2008). Handbook of Semiconductor Manufacturing Technology.

[6] Gupta B., Hossain M.A., Riaz A., Sharma A., Zhang D., Tan H.H., Jagadish C., Catchpole K., Hoex B., Karuturi S. (2022). Recent Advances in Materials Design Using Atomic Layer Deposition for Energy Applications. Adv. Funct. Mater..

[7] Zhang J., Li Y., Cao K., Chen R. (2022). Advances in Atomic Layer Deposition. Nanomanuf. Metrol..

[8] Shahmohammadi M., Mukherjee R., Sukotjo C., Diwekar U.M., Takoudis C.G. (2022). Recent Advances in Theoretical Development of Thermal Atomic Layer Deposition: A Review. Nanomaterials.

[9] Tsai Y., Li Z., Hu S. (2022). Recent Progress of Atomic Layer Technology in Spintronics: Mechanism, Materials and Prospects. Nanomaterials.

[10] Hashemi F.S.M., Prasittichai C., Bent S.F. (2015). Self-Correcting Process for High Quality Patterning by Atomic Layer Deposition. Acs Nano.

[11] Chen R., Bent S.F. (2006). Chemistry for Positive Pattern Transfer Using Area-Selective Atomic Layer Deposition. Adv. Mater..

[12] Hashemi F.S.M., Bent S.F. (2016). Sequential Regeneration of Self-Assembled Monolayers for Highly Selective Atomic Layer Deposition. Adv. Mater. Interfaces.

[13] Fang M., Ho J.C. (2015). Area-Selective Atomic Layer Deposition: Conformal Coating, Subnanometer Thickness Control, and Smart Positioning. Acs Nano.

[14] Hashemi F.S.M., Birchansky B.R., Bent S.F. (2016). Selective Deposition of Dielectrics: Limits and Advantages of Alkanethiol Blocking Agents on Metal-Dielectric Patterns. ACS Appl. Mater. Interfaces.

[15] Seo S., Yeo B.C., Han S.S., Yoon C.M., Yang J.Y., Yoon J., Yoo C., Kim H.J., Lee Y.B., Lee S.J. (2017). Reaction Mechanism of Area-Selective Atomic Layer Deposition for Al2O3 Nanopatterns. ACS Appl. Mater. Interfaces.

[16] Mackus A.J.M., Merkx M.J.M., Kessels W.M.M. (2019). From the Bottom-Up: Toward Area-Selective Atomic Layer Deposition with High Selectivity. Chem. Mater..

[17] Bobb-Semple D., Nardi K.L., Draeger N., Hausmann D.M., Bent S.F. (2019). Area-Selective Atomic Layer Deposition Assisted by Self-Assembled Monolayers: A Comparison of Cu, Co, W., and Ru. Chem. Mater..

[18] Yarbrough J., Shearer A.B., Bent S.F. (2021). Next generation nanopatterning using small molecule inhibitors for area-selective atomic layer deposition. J. Vac. Sci. Technol. A.

[19] Avila J.R., DeMarco E.J., Emery J.D., Farha O.K., Pellin M.J., Hupp J.T., Martinson A.B.F. (2014). Real-Time Observation of Atomic Layer Deposition Inhibition: Metal Oxide Growth on Self-Assembled Alkanethiols. ACS Appl. Mater. Interfaces.

[20] Hashemi F.S.M., Prasittichai C., Bent S.F. (2014). A New Resist for Area Selective Atomic and Molecular Layer Deposition on Metal-Dielectric Patterns. J. Phys. Chem. C.

[21] Chen R., Kim H., McIntyre P.C., Bent S.F. (2005). Investigation of self-assembled monolayer resists for hafnium dioxide atomic layer deposition. Chem. Mater..

[22] Liu T.-L., Harake M., Bent S.F. (2023). Sequential Use of Orthogonal Self-Assembled Monolayers for Area-Selective Atomic Layer Deposition of Dielectric on Metal. Adv. Mater. Interfaces.

[23] Yarbrough J., Pieck F., Grigjanis D., Oh I.-K., Maue P., Tonner-Zech R., Bent S.F. (2022). Tuning Molecular Inhibitors and Aluminum Precursors for the Area-Selective Atomic Layer Deposition of Al2O3. Chem. Mater..

[24] Yarbrough J., Pieck F., Shearer A.B., Maue P., Tonner-Zech R., Bent S.F. (2023). Area-Selective Atomic Layer Deposition of Al2O3 with a Methanesulfonic Acid Inhibitor. Chem. Mater..

[25] Liu T.-L., Zeng L., Nardi K.L., Hausmann D.M., Bent S.F. (2021). Characterizing Self-Assembled Monolayer Breakdown in Area-Selective Atomic Layer Deposition. Langmuir.

[26] Kessels W.M.M., Knoops H.C.M., Dielissen S.A.F., Mackus A.J.M., van de Sanden M.C.M. (2009). Surface reactions during atomic layer deposition of Pt derived from gas phase infrared spectroscopy. Appl. Phys. Lett..

[27] Kwon J., Dai M., Halls M.D., Langereis E., Chabal Y.J., Gordon R.G. (2009). In Situ Infrared Characterization during Atomic Layer Deposition of Lanthanum Oxide. J. Phys. Chem. C.

[28] Langereis E., Keijmel J., van de Sanden M.C.M., Kessels W.M.M. (2008). Surface chemistry of plasma-assisted atomic layer deposition of Al_2_O_3_ studied by infrared spectroscopy. Appl. Phys. Lett..

[29] Gong B., Parsons G.N. (2012). Quantitative in situ infrared analysis of reactions between trimethylaluminum and polymers during Al_2_O_3_ atomic layer deposition. J. Mater. Chem..

[30] Sperling B.A., Kimes W.A., Maslar J.E. (2010). Reflection absorption infrared spectroscopy during atomic layer deposition of HfO_2_ films from tetrakis(ethylmethylamido)hafnium and water. Appl. Surf. Sci..

[31] Bobb-Semple D., Zeng L., Cordova I., Bergsman D.S., Nordlund D., Bent S.F. (2020). Substrate-Dependent Study of Chain Orientation and Order in Alkylphosphonic Acid Self-Assembled Monolayers for ALD Blocking. Langmuir.

[32] Liu T.-L., Bent S.F. (2021). Area-Selective Atomic Layer Deposition on Chemically Similar Materials: Achieving Selectivity on Oxide/Oxide Patterns. Chem. Mater..

[33] Mendelsohn R., Mao G., Flach C.R. (2010). Infrared reflection–absorption spectroscopy: Principles and applications to lipid–protein interaction in Langmuir films. Biochim. Biophys. Acta (BBA)-Biomembr..

[34] Monyoncho E.A., Zamlynny V., Woo T.K., Baranova E.A. (2018). The utility of polarization modulation infrared reflection absorption spectroscopy (PM-IRRAS) in surface and in situ studies: New data processing and presentation approach. Analyst.

[35] Raval R. (1995). Probing the nature of molecular chemisorption using RAIRS. Surf. Sci..

[36] Trenary M. (2000). Reflection Absorption Infrared Spectroscopy and the Structure of Molecular Adsorbates on Metal Surfaces. Annu. Rev. Phys. Chem..

[37] Fan J., Trenary M. (1994). Symmetry and the Surface Infrared Selection Rule for the determination of the Structure of Molecules on Metal Surfaces. Langmuir.

[38] Allara D.L., Baca A., Pryde C.A. (1978). Distortions of Band Shapes in External Reflection Infrared Spectra of Thin Polymer Films on Metal Substrates. Macromolecules.

[39] Li B., Sullivan T.D., Lee T.C., Badami D. (2004). Reliability challenges for copper interconnects. Microelectron. Reliab..

[40] Li Z., Tian Y., Teng C., Cao H. (2020). Recent Advances in Barrier Layer of Cu Interconnects. Materials.

[41] Bekiaris N., Wu Z., Ren H., Naik M., Park J.H., Lee M., Ha T.H., Hou W., Bakke J.R., Gage M. Cobalt fill for advanced interconnects. Proceedings of the 2017 IEEE International Interconnect Technology Conference (IITC).

[42] Cheng Y.-L., Huang H.-C., Lee C.-Y., Chen G.-S., Fang J.-S. (2020). Comparison of Cu and Co Integration with Porous Low-k SiOCH Dielectrics. Thin Solid Film..

[43] Croes K., Adelmann C., Wilson C.J., Zahedmanesh H., Pedreira O.V., Wu C., Leśniewska A., Oprins H., Beyne S., Ciofi I. Interconnect metals beyond copper: Reliability challenges and opportunities. Proceedings of the 2018 IEEE International Electron Devices Meeting (IEDM).

[44] Wen L.G., Roussel P., Pedreira O.V., Briggs B., Groven B., Dutta S., Popovici M.I., Heylen N., Ciofi I., Vanstreels K. (2016). Atomic Layer Deposition of Ruthenium with TiN Interface for Sub-10 nm Advanced Interconnects beyond Copper. ACS Appl. Mater. Interfaces.

[45] Katzenmeyer A.M., Holland G., Chae J., Band A., Kjoller K., Centrone A. (2015). Mid-infrared spectroscopy beyond the diffraction limit via direct measurement of the photothermal effect. Nanoscale.

[46] Xu X.G., Rang M., Craig I.M., Raschke M.B. (2012). Pushing the Sample-Size Limit of Infrared Vibrational Nanospectroscopy: From Monolayer toward Single Molecule Sensitivity. J. Phys. Chem. Lett..

[47] Nowak D., Morrison W., Wickramasinghe H.K., Jahng J., Potma E., Wan L., Ruiz R., Albrecht T.R., Schmidt K., Frommer J. (2016). Nanoscale chemical imaging by photoinduced force microscopy. Sci. Adv..

[48] Centrone A. (2015). Infrared Imaging and Spectroscopy Beyond the Diffraction Limit. Annu. Rev. Anal. Chem..

[49] Dazzi A., Prater C.B. (2017). AFM-IR: Technology and Applications in Nanoscale Infrared Spectroscopy and Chemical Imaging. Chem. Rev..

[50] Rikanati L., Dery S., Gross E. (2021). AFM-IR and s-SNOM-IR measurements of chemically addressable monolayers on Au nanoparticles. J. Chem. Phys..

[51] Morsch S., Lyon S., Edmondson S., Gibbon S. (2020). Reflectance in AFM-IR: Implications for Interpretation and Remote Analysis of the Buried Interface. Anal. Chem..

[52] Nakamoto K. (1986). Infrared and Raman Spectra of Inorganic and Coordination Compounds.

[53] Deacon G.B., Phillips R.J. (1980). Relationships between the carbon-oxygen stretching frequencies of carboxylato complexes and the type of carboxylate coordination. Coord. Chem. Rev..

[54] Zeleňák V., Vargová Z., Györyová K. (2007). Correlation of infrared spectra of zinc(II) carboxylates with their structures. Spectrochim. Acta Part A Mol. Biomol. Spectrosc..

[55] López-González L.E., Guerrero-Sánchez J., Tiznado H. (2023). Soft removal of stearic acid self-assembled monolayer for area-selective atomic layer deposition. Surf. Interfaces.

[56] Sreedharan R., Mohan M., Saini S., Roy A., Bhattacharjee K. (2021). Intermediate Cu-O-Si Phase in the Cu-SiO_2_/Si(111) System: Growth, Elemental, and Electrical Studies. ACS Omega.

[57] Wang S.-Q. (1994). Barriers Against Copper Diffusion into Silicon and Drift Through Silicon Dioxide. MRS Bull..

[58] McBrayer J.D., Swanson R.M., Sigmon T.W. (1986). Diffusion of Metals in Silicon Dioxide. J. Electrochem. Soc..

[59] Hozawa K., Yugami J. (2004). Copper Diffusion Behavior in SiO_2_/Si Structure During 400 °C Annealing. Jpn. J. Appl. Phys..

[60] Kotilainen M., Vuoristo P. (2016). Aluminium and tantalum nitride barriers against copper diffusion in solar absorbers. Surf. Eng..

[61] Al-Nayili A., Majdi H.S., Albayati T.M., Saady N.M.C. (2022). Formic Acid Dehydrogenation Using Noble-Metal Nanoheterogeneous Catalysts: Towards Sustainable Hydrogen-Based Energy. Catalysts.

[62] Yang W., Lu Y., Zhou C., Zhang J., Suga T. (2018). Study of Cu Film Surface Treatment Using Formic Acid Vapor/Solution for Low Temperature Bonding. J. Electrochem. Soc..

[63] Iglesia E., Boudart M. (1983). Decomposition of formic acid on copper, nickel, and copper-nickel alloys: II. Catalytic and temperature-programmed decomposition of formic acid on CuSiO2, CuAl2O3, and Cu powder. J. Catal..

[64] Bowker M., Rowbotham E., Leibsle F.M., Haq S. (1996). The adsorption and decomposition of formic acid on Cu {110}. Surf. Sci..

[65] Lim M.S., Feng K., Chen X., Wu N., Raman A., Nightingale J., Gawalt E.S., Korakakis D., Hornak L.A., Timperman A.T. (2007). Adsorption and Desorption of Stearic Acid Self-Assembled Monolayers on Aluminum Oxide. Langmuir.

[66] Kimura F., Umemura J., Takenaka T. (1986). FTIR-ATR studies on Langmuir-Blodgett films of stearic acid with 1–9 monolayers. Langmuir.

[67] Kashid S.M., Bagchi S. (2014). Experimental Determination of the Electrostatic Nature of Carbonyl Hydrogen-Bonding Interactions Using IR-NMR Correlations. J. Phys. Chem. Lett..

[68] Fried S.D., Bagchi S., Boxer S.G. (2013). Measuring Electrostatic Fields in Both Hydrogen-Bonding and Non-Hydrogen-Bonding Environments Using Carbonyl Vibrational Probes. J. Am. Chem. Soc..

[69] Moltved K.A., Kepp K.P. (2019). The Chemical Bond between Transition Metals and Oxygen: Electronegativity, d-Orbital Effects, and Oxophilicity as Descriptors of Metal–Oxygen Interactions. J. Phys. Chem. C.

[70] Kepp K.P. (2016). A Quantitative Scale of Oxophilicity and Thiophilicity. Inorg. Chem..

[71] Luschtinetz R., Oliveira A.F., Duarte Hélio A., Seifert G. (2010). Self-assembled Monolayers of Alkylphosphonic Acids on Aluminum Oxide Surfaces—A Theoretical Study. Z. Anorg. Allg. Chem..

