# Supplementary material for: Stearic Acid as an Atomic Layer Deposition Inhibitor: Spectroscopic Insights from AFM-IR
Source: Nanomaterials (Basel). 2023 Oct 6;13(19):2713. doi: 10.3390/nano13192713 (PMC10574727; doi:10.3390/nano13192713)
Supplement: Supplementary file 1 [file nanomaterials-13-02713-s001.zip › nanomaterials-2590531-supplementary.pdf]

Supporting Information:

Stearic Acid as an Atomic Layer Deposition Inhibitor: Spectroscopic Insights from AFM-IR

Saumya Satyarthy [1,†], Md Hasan Ul Iqbal [1,†], Fairoz Abida [2], Ridwan Nahar [3], Adam J. Hauser [3], Mark Ming-Cheng Cheng [2] and Ayanjeet Ghosh [1,*]

[1] Department of Chemistry and Biochemistry, The University of Alabama, Tuscaloosa, AL 35487, USA; ssatyarthy@crimson.ua.edu (S.S.); mhasanuliqbal@crimson.ua.edu (M.H.U.I.)
[2] Department of Electrical and Computer Engineering, The University of Alabama, Tuscaloosa, AL 35487, USA; fabida@crimson.ua.edu (F.A.); mmcheng@eng.ua.edu (M.M.-C.C.)
[3] Department of Physics and Astronomy, The University of Alabama, Tuscaloosa, AL 35487, USA; rnahar1@crimson.ua.edu (R.N.); ahauser@ua.edu (A.J. H.)
[*] Correspondence: ayanjeet.ghosh@ua.edu
[†] The authors contributed equally to this work.

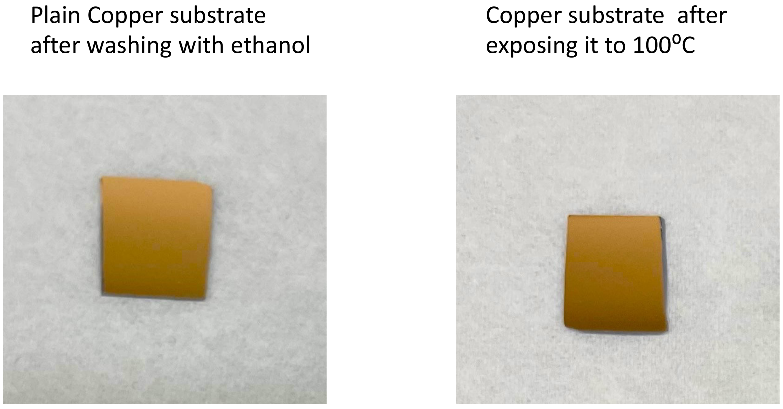

Figure S1. Optical images of bare Cu substrates before (left) and after (right) heating at 100 °C, indicating no visual signs of degradation.

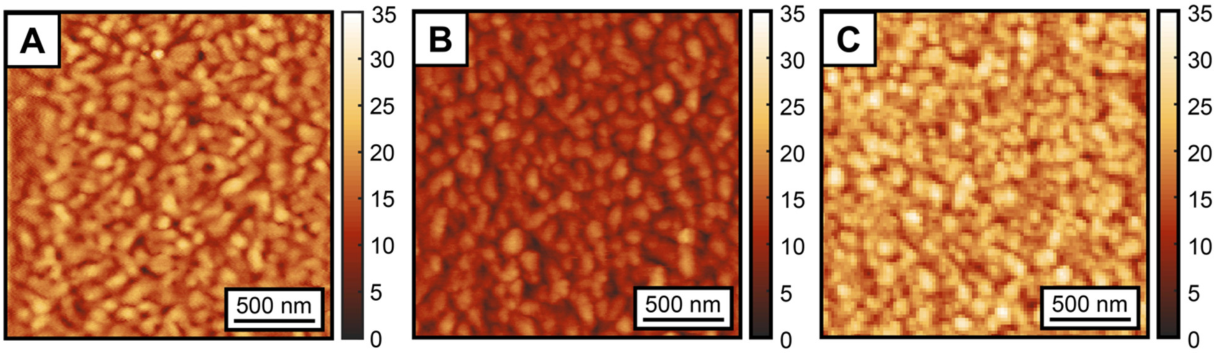

Figure S2. AFM topographs of SA SAMs on Cu after A) 25 cycles, B) 50 cycles and C) 200 cycles of ALD.

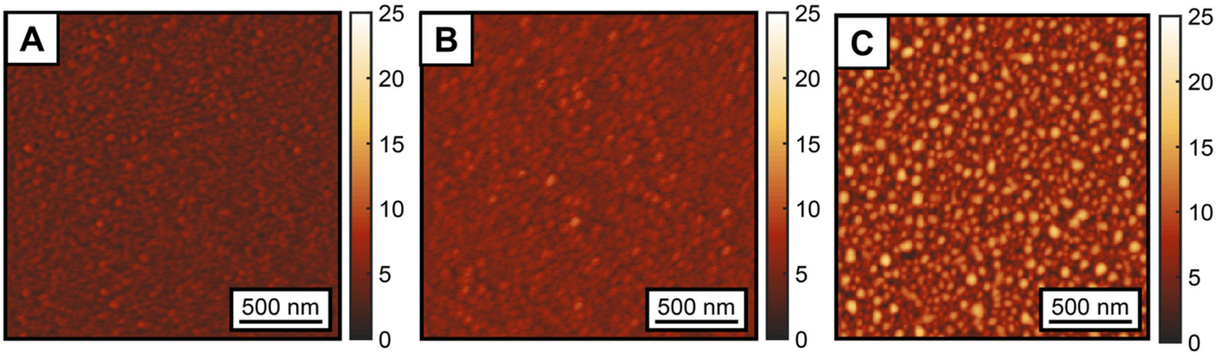

Figure S3. AFM topographs of SA SAMs on Co after A) 25 cycles, B) 50 cycles and C) 200 cycles of ALD.

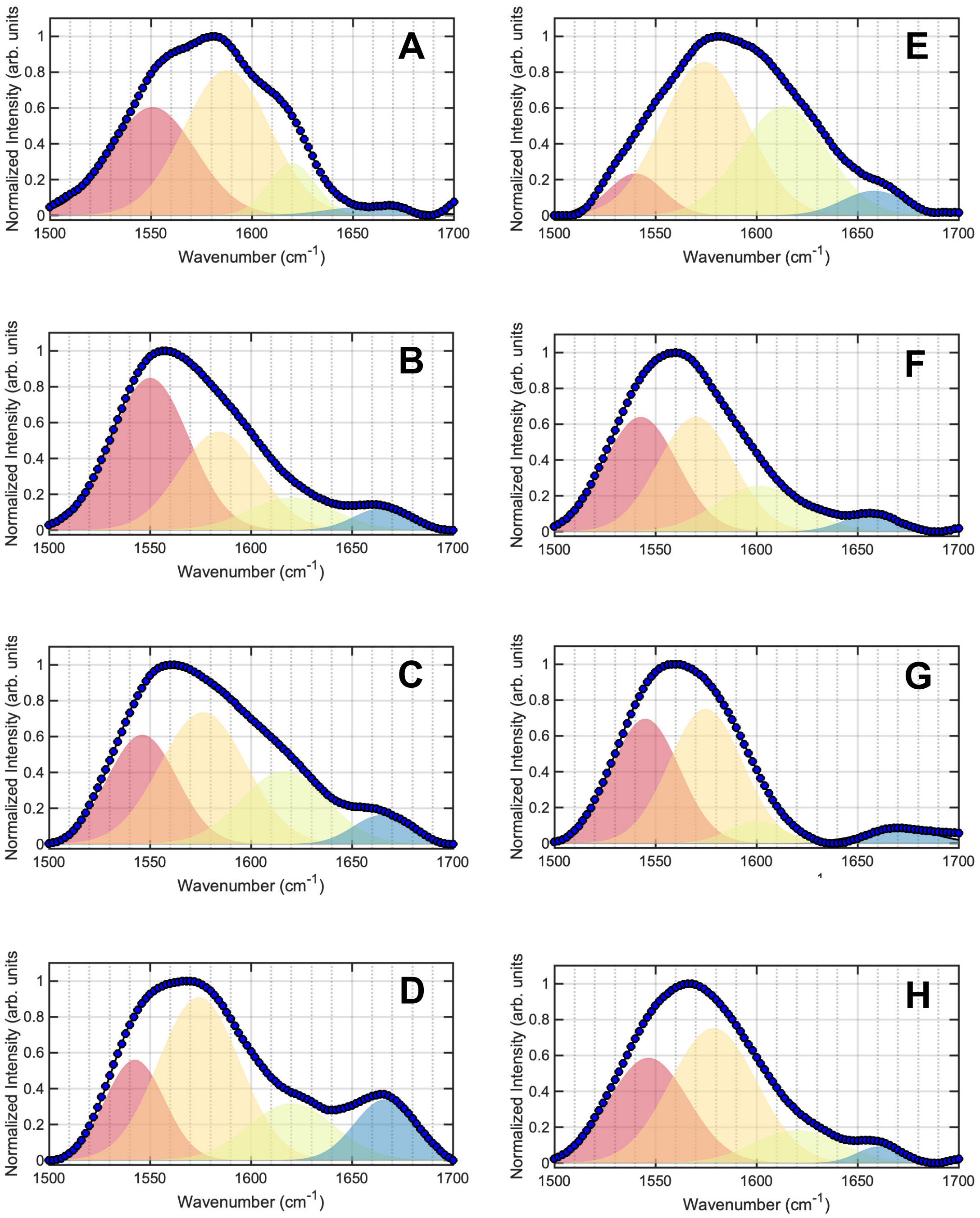

Figure S4. Curve fitting of AFM-IR spectra of SA SAMs corresponding to A) 0 cycles ALD on Cu, B) 25 cycles ALD on Cu, C) 50 cycles ALD on Cu, D) 200 cycles ALD on Cu, E) 0 cycles ALD on Co, F) 25 cycles ALD on Co, G) 50 cycles ALD on Co and H) 200 cycles ALD on Co.
